# Are dispersion corrections accurate outside equilibrium? A case study on benzene

**DOI:** 10.3762/bjoc.14.99

**Published:** 2018-05-23

**Authors:** Tim Gould, Erin R Johnson, Sherif Abdulkader Tawfik

**Affiliations:** 1 Queensland Micro- and Nanotechnology Centre, Griffith University, Nathan, Queensland 4111, Australia; 2 Department of Chemistry, Dalhousie University, Halifax, Nova Scotia, B3H 4R2, Canada; 3 School of Mathematical and Physical Sciences, University of Technology Sydney, Ultimo, New South Wales 2007, Australia; 4 Institute for Biomedical Materials and Devices (IBMD), Faculty of Science, University of Technology Sydney, Sydney, NSW, Australia

**Keywords:** benzene, DFT, dispersion, van der Waals

## Abstract

Modern approaches to modelling dispersion forces are becoming increasingly accurate, and can predict accurate binding distances and energies. However, it is possible that these successes reflect a fortuitous cancellation of errors at equilibrium. Thus, in this work we investigate whether a selection of modern dispersion methods agree with benchmark calculations across several potential-energy curves of the benzene dimer to determine if they are capable of describing forces and energies outside equilibrium. We find the exchange-hole dipole moment (XDM) model describes most cases with the highest overall agreement with reference data for energies and forces, with many-body dispersion (MBD) and its fractionally ionic (FI) variant performing essentially as well. Popular approaches, such as Grimme-D and van der Waals density functional approximations (vdW-DFAs) underperform on our tests. The meta-GGA M06-L is surprisingly good for a method without explicit dispersion corrections. Some problems with SCAN+rVV10 are uncovered and briefly discussed.

## Introduction

The past decade has seen an increasing awareness of the role played by van der Waals dispersion forces in chemistry and materials science [1–6]. It has consequently become firmly established that including dispersion forces can be vital for understanding and predicting the behaviour and structure of molecules, materials and surfaces [7–12].

The increased attention being paid to dispersion forces has paralleled, and been driven by, an increased interest in how to accurately model them. Multiple families of approaches for including dispersion forces in quantum chemical simulations now exist, mostly based around the principle of improving density functional theory (DFT) calculations (see, e.g., some key and recent summaries [3,5,13–14]) through a dispersion correction. The latest variants of these approaches have been highly successful in predicting key properties of a wide range of molecules and materials, such as binding energies and molecular/material structures [4–5,15]. Methods are increasingly converging towards accurate prediction of these properties [16].

What is less well known, however, is how well methods predict the properties of systems outside of internal equilibrium, i.e., whether they can predict energies and forces when a system has not relaxed to its lowest energy geometry. This question is important as it is feasible that methods benefit from a cancellation of errors at equilibrium, which may give false expectations about their general accuracy. We therefore need to understand the limitations of approaches in dealing with dispersion forces generally, and not just for systems at their equilibrium geometries. This is especially important for predicting how a system (or sub-system) behaves when subject to external forces, or when dispersion forces compete with other weak forces within molecules or structures. It is particularly relevant as recent work has shown that modern approaches can often provide accurate binding distances or binding energies in layered materials [17], but not both, suggesting limits to their accuracy.

The work by Řezáč et al. goes some way to resolving this question, by providing benchmark values (the S66x8 set) for eight equilibrium and non-equilibrium geometries of different molecular pairs [18]. This set has been used to test various dispersion methods [19]. However, while S66x8 certainly improves on tests only at optimized geometries, it may still fail to expose issues with forces or other energetic differences.

In this work we thus test the accuracy of modern dispersion approaches in reproducing the energetics of the benzene dimer, an important model system, in different geometries. The coupled-cluster with singles, doubles and perturbative triples [CCSD(T)] benchmark set of Sinnokrot et al. [20] is used, with the aim of establishing which approximations can best reproduce the full potential energy curves. This simple test is not designed to be comprehensive, but rather to interrogate the predictive ability of different approaches. Note that the benchmark set, while slightly inaccurate by modern standards, is predicted to be within 0.1 kcal/mol of more recent benchmarks [21], which is similar to methodological errors caused by using modern pseudopotential methods [22]. We thus feel that its range more than makes up for any limitations it may have.

Moreover, interactions between ring structures feature widely across organic chemistry [23–25]. Recently, there has been increasing interest in utilizing non-covalent π-stacking for synthetic catalysis – and it is notable that most structures shown in a recent review on the topic feature rings that interact at distances greater than the potential minimum [26]. Benzene dimers also feature in the S22 benchmark set [21,27] that is often used to semi-empirically optimize dispersion corrections, and is almost always used as a test of such methods. They are thus an excellent test of the quality of dispersion models on a system where failures may have chemical relevance.

## Results and Discussion

### The origin of dispersion forces

Dispersion forces are a semi-classical effect coming from quantum fluctuations. Most simply, they can be viewed from the perspective of pairs of interacting fluctuating dipoles, in which a temporary dipole in one (sub)system induces a dipole in the other, and consequently lowers the total energy. Since the field from each temporary dipole falls off as the inverse cube of the distance *D* between the systems, and the contributions come in pairs, this leads to an interaction with the asymptotic form *U*_vdW_ = −*C*_6_*D*^−6^, where *C*_6_ is a coefficient that depends on the properties of the independent systems. A similar semi-classical analysis can also be applied to more general multipoles, such as quadropoles, which give rise to terms proportional to *D*^−8^ (quadropole with dipole), *D*^−10^ (quadrupole–quadropole) etc.

In addition to direct coupling between pairs of multipoles, more general forms of coupling can also lead to higher order contributions, including 3rd-order effects between three multipoles, 4th-order etc., as detailed in, e.g., Dobson and Gould [2]. In certain cases, including graphene [28–31], this can lead to fundamental deviations from the simple model outlined above [18,31–36]. However, the most extreme deviations from the pairwise model do not affect the benzene dimer system.

Note, the importance of higher-order (many-body) dispersion terms in “typical” systems has been the subject of some debate. It is critical to differentiate between non-additive many-body electronic interactions [34–35] and non-additive *C*_9_ or Axilrod-Teller-Muto (ATM) dispersion interactions here. The former cause large differences in the effective pairwise *C*_6_ and higher-order dispersion coefficients, relative to corresponding values for free atoms [33,37–38] (these are known as Type-B non-additivity effects in the classification scheme of Dobson [33]). This is a particularly significant effect for metals and can alter the *C*_6_ coefficients by more than an order of magnitude in some cases [39–40]. In contrast, the 3-body *C*_9_ contribution to the dispersion energy is typically smaller in magnitude than the pairwise *C*_10_ contribution [41] and consequently is negligible for most applications compared to 3-body electronic effects [42–44].

Mathematically, the ATM treatment is most applicable when the energy contribution from 3rd and higher order terms converge rapidly as a function of inverse distance and may thus be truncated after the 2nd-order or 3rd-order contributions. Many-body effects arise from a divergence or slow convergence in the same series due to Dobson-B or -C effects, so that the contributions must be treated as a formal power series and rewritten as an explicit function of the polarisability tensor.

### Summary of modern dispersion methods

Over the last decade, a number of new approaches have been developed that explicitly introduce dispersion forces into electronic structure theory methods – typically density functional theory (DFT). These approaches seek to overcome the fundamental lack of dispersion forces in DFT and Hartree–Fock theory by introducing an explicit long-range correction, giving a total energy *E* = *E*_DFT_ + Δ*E*_vdW_ for the system. Typically, *E*_DFT_ is taken from a standard density functional approximation (DFA), such as PBE [45] or B3LYP [37], while Δ*E*_vdW_ is one of a range of dispersion correction models. Note, this is different to seamless approaches like MP2, RPA or other quantum chemistry methods which include dispersion forces automatically.

Common van der Waals corrections can be broadly divided into three categories, as will be detailed below. Substantial effort has seen steady improvements in the quality of approaches in all three categories. In this paper we focus only on recent (or older, but still very popular) iterations within each category, to reflect how the methods are designed to be used in practice. The three classes of approaches considered are:

1) Purely empirical corrections based only on semi-classical models of the nuclei, and their neighbours, without drawing from the electronic density [15,46–50]. Of these we include Grimme’s D2, D3 and D3-BJ functionals, as corrections on PBE and, in the case of D3-BJ, on B3LYP. Here and henceforth “on X” means that the correction is taken on top of the X (hybrid) density functional approximation, which we also denote as X-Y (e.g., PBE-D3-BJ);

2) atomic-dipole with density methods, which correct first-principles or empirical models of atomic dipoles (and sometimes multipoles) using properties of the electronic density. Of these we include XDM [51–52] (on various DFAs), TS [53] (on PBE), TS-MBD@rsRSC (MBD for short, on PBE) and FI-MBD@rsRSC (FI for short, on PBE). Both MBD [54–55] and FI [56–57] include dispersion contributions to all orders using the many-body dispersion method of Tkatchenko et al. [54], but involve different treatment of polarisabilities and screening; and

3) first principles density functionals, in which dispersion forces depend only on the density in a totally seamless fashion [58–60] and in which the base DFA forms part of the functional itself. We include the functional of Dion et al. [59], vdW-DF2 [61], optPBEvdW [62] and optB88vdW [62]. We also include SCAN+rVV10, based on the strongly-constrained and normalised (SCAN) meta-GGA, which has been shown to be very successful in initial testing [63]. We refer collectively to these as vdW-DFAs.

In addition to the van der Waals functionals, we also show energies from the generalized gradient approximations (GGAs) PBE and B3LYP, and the meta-GGAs M06L [64] and SCAN [65] without dispersion corrections. Although both GGAs are known to neglect dispersion physics, the meta-GGAs M06-L and SCAN are expected to capture some dispersion-binding contributions through the large-gradient behaviour of their exchange functionals. Note, the interplay between exchange-correlation functionals and dispersion corrections has been the topic of some discussion [66–67]. Finally, we note that we include only general functionals, and avoid approaches that are designed to address one type of system (e.g., molecules, bulks or layered structures) only.

### Calculation details

We performed most calculations using VASP 5.4 [68–69] where the valence electrons are separated from the core by use of projector-augmented wave pseudopotentials (PAW) [70]. The energy tolerance for the electronic structure determinations was set at 10^−7^ eV. Calculations used only the Γ ***k***-point. ENCUT was set to 400 eV in all calculations, which were carried out in a 15 × 15 × 25 Å^3^ supercell. SCAN(+rVV10) required ENCUT = 700 eV as results showed significant noise with the standard energy cutoff, which led us to reduce the box dimensions to 12 × 12 × 20 Å^3^. We will discuss issues with SCAN later. Both MBD approaches (TS-MBD and FI-MBD) used the reciprocal space implementation [71], the latter in a custom version of VASP 5.4.1 [57]. The vdW-DFs use the implementation of Klimeš [72] of the Román-Pérez and Soler [73] method.

Some methods are not implemented in VASP and in these cases, the calculations were performed using other codes. XDM results were obtained using Gaussian09 (PBE, B3LYP, and LC-wPBE) or Psi4 [74] (B86bPBE) and the postg application. We include XDM results on several base functionals due to its broad success. M06-L results were calculated with Gaussian16 using the aug-cc-pVTZ basis set due to convergence difficulties with the plane-waves/pseudopotential approach.

To put these settings in context, we purposefully employed the methods as they are intended to be used, i.e., using more-or-less standard convergence parameters and recommended settings.

## Results

**Figure 1:**
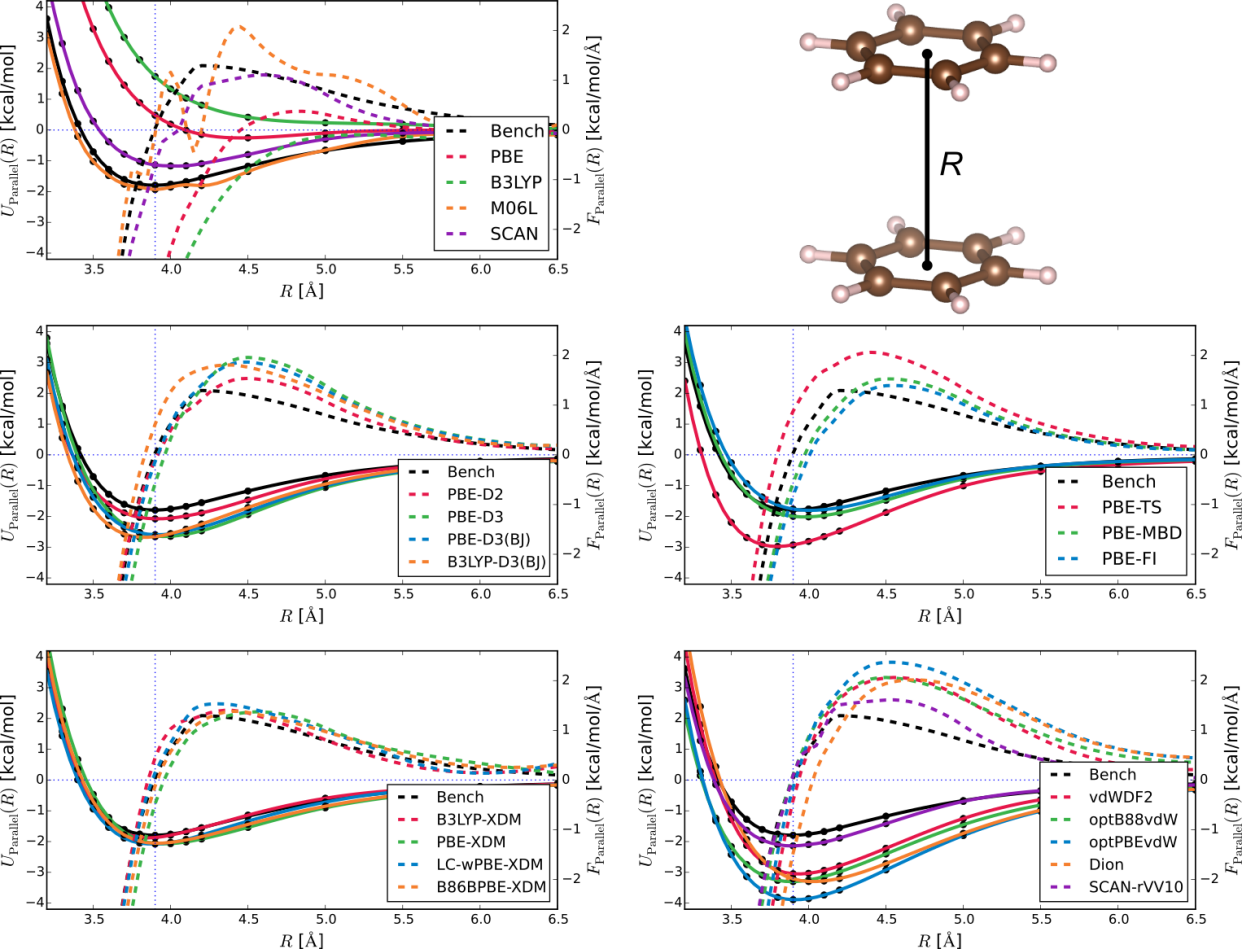
Interaction energies (solid lines) and forces (dashed lines) for the parallel configuration of the benzene dimer. Each panel groups a different family of computational approach. The top row (from left to right) shows GGAs and meta-GGAs without dispersion corrections, and the dimer geometry. The second row reports Grimme-D variants (l) and TS/MBD variants (r). The bottom row shows XDM on different DFAs (l), and vdW-DFAs (r). The benchmark data is always shown in black.

**Figure 2:**
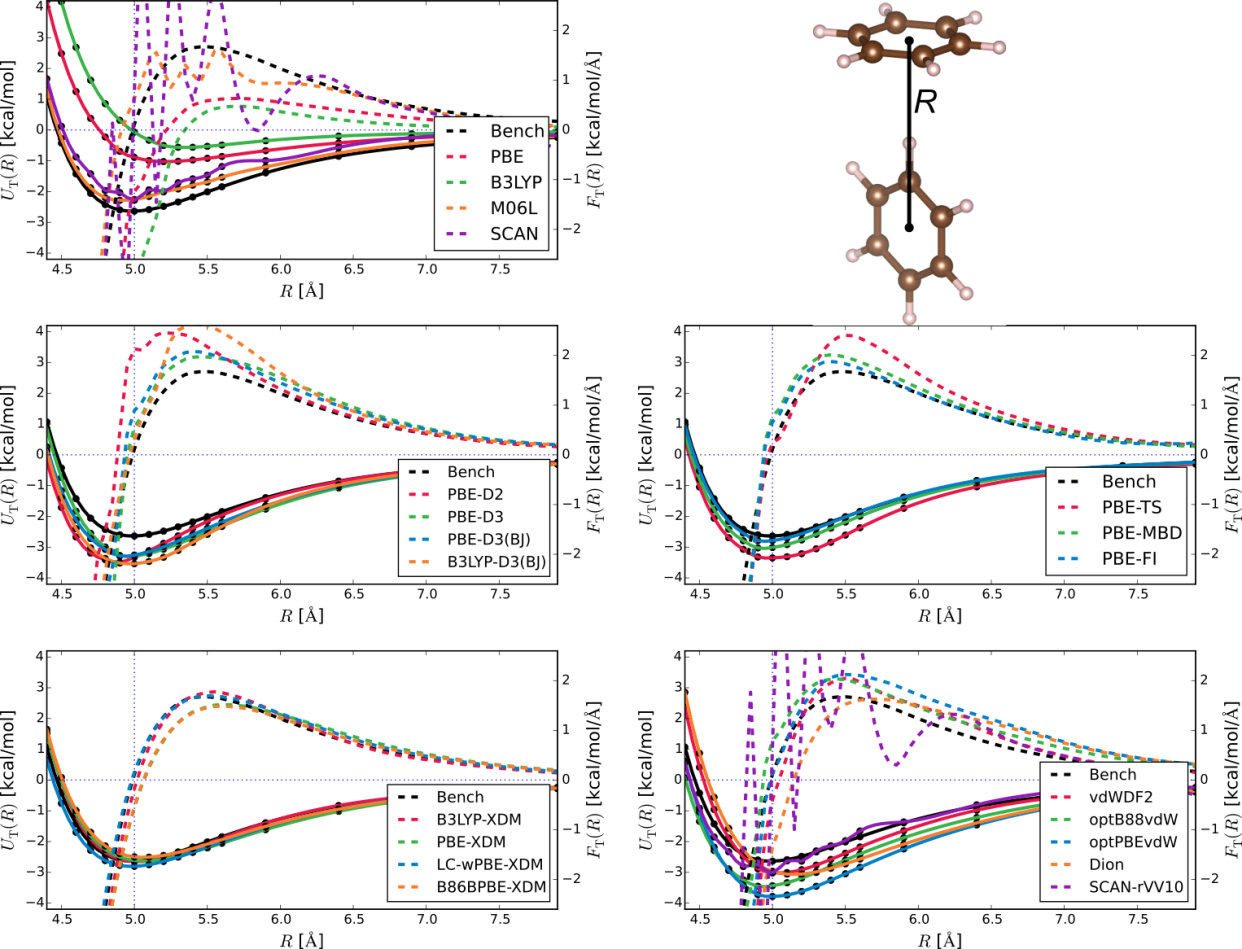
Interaction energies (solid lines) and forces (dashed lines) for the T configuration of the benzene dimer. Panels are the same as in Figure 1.

**Figure 3:**
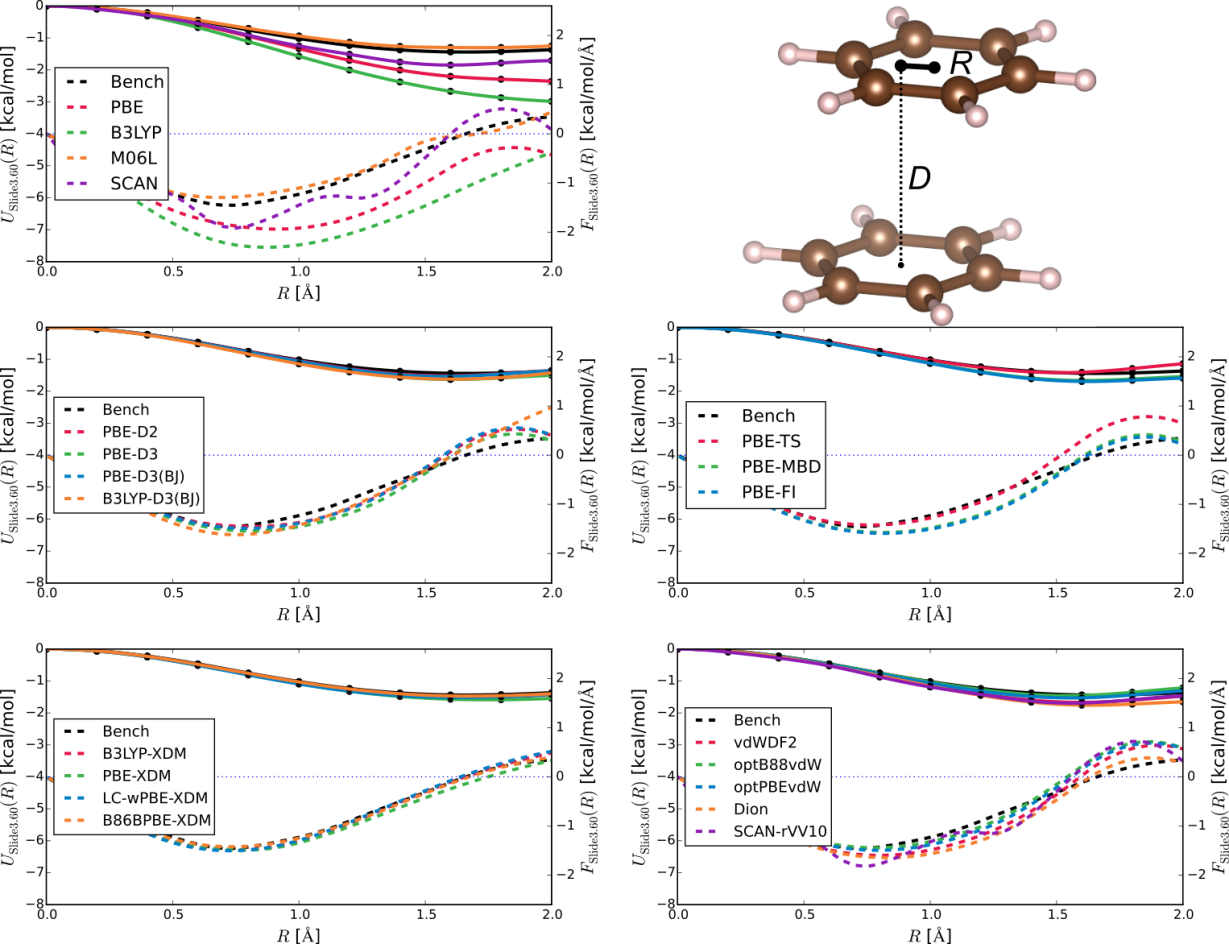
Interaction energies *U*(*R*;*D*) = *E*(*R*;*D*) − *E*(0;*D*) (solid lines) and forces (dashed lines) for the slipped-parallel configuration of the benzene dimer at *D* = 3.6 Å. Panels are the same as in Figure 1.

Now that we have established the background methodology, let us summarise the shared features of Figures 1–3 to aid in detailed assessment. Each figure is composed of sub-figures showing results for selected groupings of methods. Each sub-figure shows as solid lines the benchmark potential energy curve *U*_bench_ and the potential energy curves from the selected methods *U*_method_. They also show the benchmark force *F*_bench_, and the forces for different methods *F*_method_, all in dashes. All energies and forces are reported as functions of distance, either between the centre of dimers (Figure 1 and Figure 2) or the sliding distance (Figure 3).

We adopt some steps to ensure all energies and forces are calculated in the same way, so as to reduce uncontrolled errors from, e.g., basis set superpositions or pseudopotentials. Firstly, we use the electronic structure codes to calculate energies *E*(*R*) directly. We then fit *E*(*R*) = *E*_∞_ – *C*_6_/*R*^6^ to the last five points of the parallel configuration data to find *E*_∞_, the extrapolated energy of two monomers, which lets us determine interaction energies *U*(*R*) = *E*(*R*) − *E*_∞_. We plot *U*(*R*) in Figure 1 and Figure 2, and use the minimum-energy values directly in Table 1 – Figure 3 shows *U*(*R*) = *E*(*R*) − *E*(0). Secondly, we obtain all forces by fitting cubic splines through the energy data, and taking the derivative of the splines.

Figure 1 shows the interaction energy for the parallel configuration of the benzene dimer (labelled P – with *D_6_*_h_ symmetry). Despite having a minimum as a function of distance between the two centres, this arrangement is unstable as the dimers wish to slide apart sideways (see later discussion on Figure 3) to reduce electrostatic effects, such as overlap of the densities of the monomers and static quadrupole–quadropole interactions, which make metastable AA graphite ≈0.23 kcal/mol/C less energetically favourable compared to AB graphite [75]. This configuration thus involves competition between dispersion forces, repulsive electrostatic forces, and other exchange and correlation effects, making it a good test of dispersion corrections.

It is clear from the figure that D2, XDM (all variants), MBD and FI all give reliable energies across the entire curve. Their forces are slightly worse, but still within 0.5 kcal/mol/Å of the reference data at reasonable intermolecular distances. The more modern Grimme variants fare worse than their older cousin, and none of the two-point vdW-DFAs work very well at all, for energies or forces, except near the minima. Indeed, most of the tested vdW-DFAs give force errors outside equilibrium that are similar in magnitude to the force itself. A notable exception is SCAN+rVV10 which is broadly on a par with XDM and TS/FI-MBD. Somewhat surprisingly, the semi-local meta-GGA M06L gives an energy curve which is also in broad agreement with the benchmark, but which fluctuates [76] making the spline-derived forces less reliable. Other dispersion-free methods are less successful, as expected.

Figure 2 then reports the energies for T configuration as a function of distance (T – with *C*_2_*_v_* symmetry), which includes the global minimum for benzene dimer interactions, or at least a local minimum that is energetically very close to it. Here the balance of energetic contributions is more strongly skewed to dispersion, and it is expected that vdW dispersion corrections should work better than for the parallel configuration shown in Figure 1.

Indeed, the successful methods for the parallel geometry (XDM, MBD, FI) seem to work very well here, reproducing the reference energies and forces to within methodological error of ≈0.1 kcal/mol. The vdW-DFAs perform slightly better than in the parallel case, as one would hope. D2, however, is quite poor despite its success in the parallel case and its more modern cousins are conversely much better. Again, M06L works well. Here, however, we notice that SCAN shows significant oscillations around the true curve, which SCAN+rVV10 inherits (to be discussed later).

Next, Figure 3 reports the potential energy curves for sliding of parallel benzene molecules relative to one another at a fixed inter-planar distance *D*, known as the slipped-parallel configuration (SP – with *C_2_*_h_ symmetry). We show results for *D* = 3.6 Å, shown relative to their energy in the perfectly parallel configuration [i.e., *U*(*R*;*D*) = *E*(*R*;*D*) − *E*(0;*D*)]. Here XDM is a stand-out, giving almost perfect agreement with the benchmarks, thus indicating its ability to simultaneously capture competing energy contributions. All other methods are much more successful here than in the previous tests, reflecting their consistency in reproducing electrostatic effects compared to dispersion interactions which are more-or-less constant across the curves. These results are replicated in other tests (not shown) at *D* = 3.2, 3.4 and 3.8 Å. Again, the SCAN and SCAN+rVV10 curves display oscillations.

**Figure 4:**
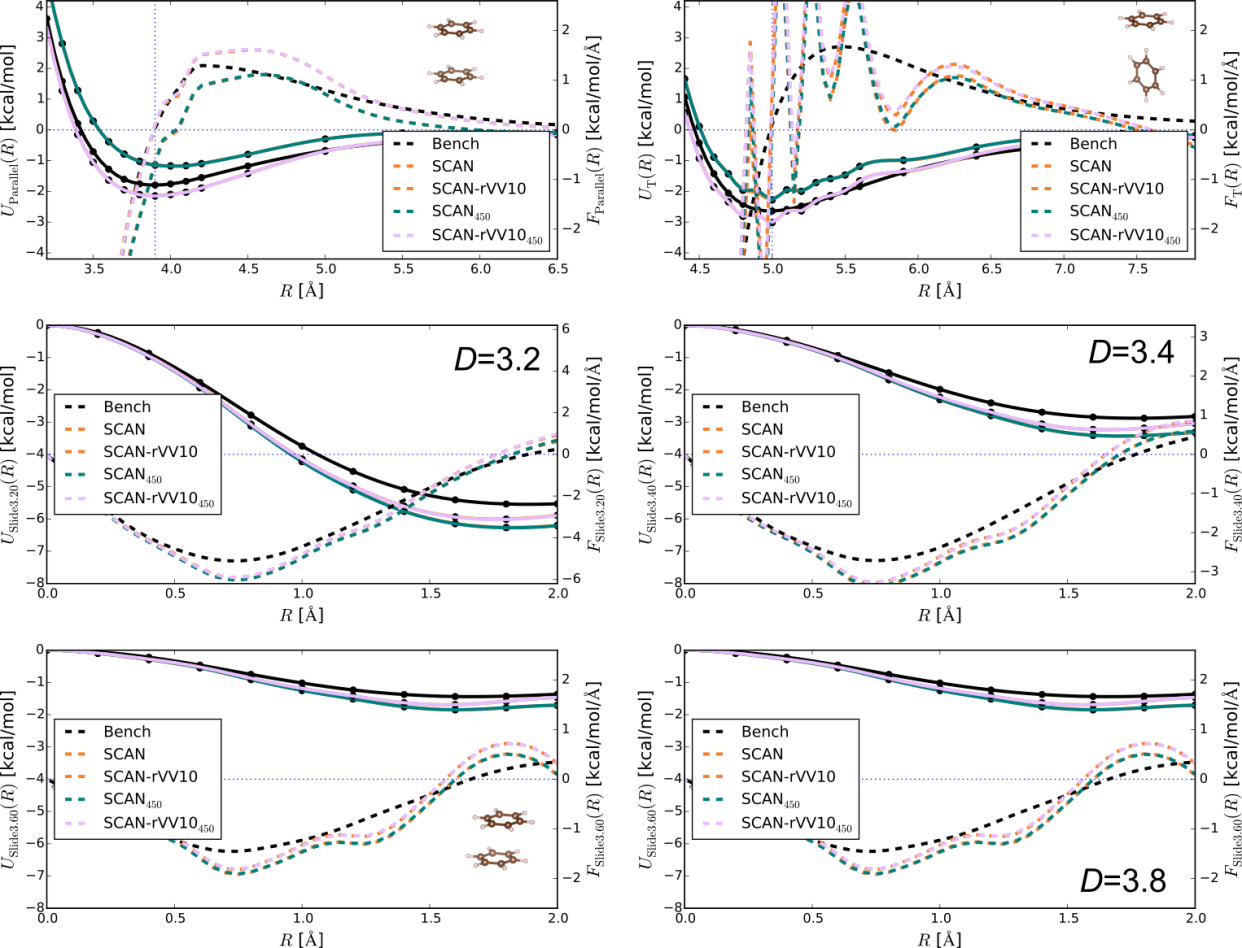
SCAN and SCAN+rVV10 results for the Parallel (top left) and T (top right) configurations of the benzene dimer, as well as for the slipped-parallel configurations at all four intermolecular distances. Results are shown for two different energy cutoffs to test convergence (the default 700 eV and a smaller cutoff of 450 eV). The panels show interaction energies (solid lines) and forces (dashed lines), as in Figure 1.

The strange behaviour of SCAN and SCAN+rVV10 warrants special attention. Previous tests of meta-GGAs using Gaussian-type orbital codes suggest this issue might be related to the density of the real-space grid [76]. In Figure 4 we thus show results for SCAN and SCAN+rVV10 for all four intermolecular distances (*D* = 3.2, 3.4, 3.6 and 3.8 Å) and for both the large energy cutoff 700 eV used in previous calculations, and also a smaller cutoff of 450 eV.

It is obvious from these curves that the oscillations seem to be smallest when the overlap between the orbitals is largest, as in the parallel case and the two closest (*D* = 3.2, 3.4) sliding cases, versus the T case and the more distant sliding cases. Furthermore, the oscillations seem to hold consistently for the smaller and larger energy cutoffs, a result we find somewhat perplexing as, if they were sensitive to the grid, we would expect them to decrease with a larger cutoff (and consequently finer grid).

Finally, in Table 1 we quantify how the different methods perform in prediction of the relative energies of the various local minima, *U*_0_(T), *U*_0_(P) and *U*_0_(SP), for the T, parallel (P) and slipped-parallel (SP) configurations, respectively. We thus show the energy differences, Δ*U*(P/SP) = *U*_0_(P/SP) − *U*_0_(T), between the local minima for the parallel and slipped-parallel configurations, and the (presumed) global minimum for the T configuration. In all cases we fit quadratic curves to data to obtain a value as close to the true minimum as possible. We also take advantage of revised benchmark values from Takatani et al. [21] to establish errors in the main reference data used for the full potential curves.

**Table 1:** Relative energy differences, Δ*U*(P/SP) = *U*_0_(P/SP) − *U*_0_(T) [in kcal/mol], between lowest energies *U*_0_(T/P/SP) for the T, parallel (P) and slipped-parallel (SP) configurations of the benzene dimer, with respect to the minimum-energy T configuration. Here we use the revised benchmarks from Takatani et al. [21] for references, and to quantify the error in our main source of benchmark data [20]. Solid lines separate the different groupings of functionals used in this paper, which are ranked within each section according to |Error|. |Error| = 1/2[|Δ*U*(P)_method_ − Δ*U*(P)_revbench_| + |Δ*U*(SP)_method_ − Δ*U*(SP)_revbench_|].

	Δ*U*(P)	Δ*U* (SP)	|Error|

revBench^a^	0.86	0.11	–
Bench^b^	0.91	−0.01	0.09

SCAN	1.33	0.27	0.32
PBE	0.95	0.82	0.40
M06L	0.51	−0.38	0.42
B3LYP	0.65	1.08	0.59

PBE-D3(BJ)	0.77	−0.16	0.18
PBE-D3	0.71	−0.11	0.19
B3LYP-D3(BJ)	0.80	−0.51	0.34
PBE-D2	1.50	0.28	0.41

PBE-FI	0.93	0.11	0.04
PBE-MBD	0.93	0.03	0.08
PBE-TS	0.43	−0.89	0.71

LC-wPBE-XDM	0.83	−0.14	0.14
B3LYP-XDM	0.83	−0.19	0.17
PBE-XDM	0.59	−0.15	0.27
B86BPBE-XDM	0.48	−0.31	0.40

SCAN-rVV10	1.03	−0.21	0.24
optB88vdW	0.18	−0.72	0.76
vdWDF2	0.06	−0.69	0.80
Dion	−0.27	−0.73	0.98
optPBEvdW	−0.12	−0.90	1.00

^a^From Takatani et al. [21], ^b^from Sinnokrot et al. [20].

Here, FI and MBD are the best-performing methods, with absolute errors smaller than those for the older benchmark data set. Variants of XDM (LC-wPBE-XDM, B3LYP-XDM) and some Grimme methods are a little poorer, but are still very good. The vdW-DFAs and PBE-TS method can be quite poor, however, further reflecting their poorer treatment of dispersion energies and forces away from equilibrium.

## Conclusion

In this work we used benchmark results for several configurations of the benzene dimer to test the ability of dispersion-corrected density functional theory to obtain accurate energies and forces away from equilibrium, and thus to understand their predictive capabilities. All the methods tested here are backed by previously reported successes on a wide range of chemical and/or materials systems. We have shown that many of them do not match these successes at equilibrium by guaranteed success outside it. In the worst cases, some methods have errors in the predicted forces as large as the force itself.

The exchange-hole dipole moment (XDM) model, which we tested with several DFAs, performs very well in general. PBE-MBD and PBE-FI (which incorporates an improved treatment of polarisabilities into an MBD-like calculation) both perform similarly well. We suspect any of these methods can be reliably trusted for predictions in systems involving benzene ring structures

Grimme’s various methods, TS theory, and various two-point van der Waals density functionals are less successful in our tests, however. M06L is surprisingly accurate for a meta-GGA without explicit dispersion corrections, but is numerically noisy [76]. We thus advise caution when using any of these methods for systems where interactions between ring structures might be important.

The results for SCAN-rVV10 are troubling. We suspect that the oscillations in the potential energy curves reflect previously reported problems with the integration grid [76]. We could, however, not remove them even with a large energy cutoff of 700 eV, just shy of the value used by its developers for rare gas solids [63]. Also, the results were very similar when a smaller energy cutoff was employed, hinting at a deeper underlying problem. This convergence issue is certainly something that should be investigated before dispersion-corrected SCAN is applied widely to weak-bonding problems.

The results reported here also strongly support the importance of using good polarizabilities (dipole and higher) in dispersion models. XDM, MBD and its FI variant include contributions from both the local density and geometry, and thus can capture type-A and -B non-additivity (the latter semi-locally in the case of XDM), in the classification scheme of Dobson [33]. By contrast, the other methods tested involve more simplistic treatment of environmental contributions to polarisabilities and dispersion coefficients.

Finally, we note that we have only tested one type of molecular dimer which means our conclusions are necessarily limited, despite the benzene dimer being an important and difficult example involving competing contributions to the interaction energies and forces. The results uncovered here are interesting enough, we feel, to establish an impetus to carry out further testing of dispersion forces away from equilibrium and to establish the role of effects beyond dipole pairs (including Axilrod–Teller–Muto terms, quadropole and higher multipole terms, and full many-body terms). We hope this work will drive development and use of new benchmarking sets, along these lines, which can test a wider range of physics and chemistry at and outside of equilibrium, and be used to improve future dispersion models.

## Supporting Information

We provide all data generated through this project to allow other researchers to carry out their own analyses. This file contains all reference data used for this paper, stored in comma separated variables format with “#” used to indicate comments.

File 1 All reference data.

## References

[1] Grimme S (2011). Wiley Interdiscip Rev: Comput Mol Sci.

[2] Dobson J F, Gould T (2012). J Phys: Condens Matter.

[3] Grimme S, Hansen A, Brandenburg J G, Bannwarth C (2016). Chem Rev.

[4] Woods L M, Dalvit D A R, Tkatchenko A, Rodriguez-Lopez P, Rodriguez A W, Podgornik R (2016). Rev Mod Phys.

[5] Hermann J, DiStasio R A, Tkatchenko A (2017). Chem Rev.

[6] Klimeš J, Michaelides A (2012). J Chem Phys.

[7] Geim A K, Grigorieva I V (2013). Nature.

[8] Rösel S, Quanz H, Logemann C, Becker J, Mossou E, Cañadillas-Delgado L, Caldeweyher E, Grimme S, Schreiner P R (2017). J Am Chem Soc.

[9] Fabrizio A, Corminboeuf C (2018). J Phys Chem Lett.

[10] Johnson E R, Clarkin O J, Dale S G, DiLabio G A (2015). J Phys Chem A.

[11] Reimers J R, Li M, Wan D, Gould T, Ford M J (2017). Non-Covalent Interactions in Quantum Chemistry and Physics.

[12] Gould T, Lebègue S, Björkman T, Dobson J F, Iacopi F, Boeckl J J, Jagadish C (2016). Semiconductors and Semimetals.

[13] Goerigk L (2017). Non-Covalent Interactions in Quantum Chemistry and Physics.

[14] Schröder E, Cooper V R, Berland K, Lundqvist B I, Hyldgaard P, Thonhauser T (2017). Non-Covalent Interactions in Quantum Chemistry and Physics.

[15] Grimme S, Ehrlich S, Goerigk L (2011). J Comput Chem.

[16] Goerigk L, Hansen A, Bauer C, Ehrlich S, Najibi A, Grimme S (2017). Phys Chem Chem Phys.

[17] Tawfik S A, Gould T, Stampfl C, Ford M J (2018). Phys Rev Materials.

[18] Řezáč J, Riley K E, Hobza P (2011). J Chem Theory Comput.

[19] Goerigk L, Kruse H, Grimme S (2011). ChemPhysChem.

[20] Sinnokrot M O, Sherrill C D (2004). J Phys Chem A.

[21] Takatani T, Hohenstein E G, Malagoli M, Marshall M S, Sherrill C D (2010). J Chem Phys.

[22] Lejaeghere K, Bihlmayer G, Björkman T, Blaha P, Blügel S, Blum V, Caliste D, Castelli I E, Clark S J, Corso A D (2016). Science.

[23] Hermann J, Alfè D, Tkatchenko A (2017). Nat Commun.

[24] Sutton C, Risko C, Brédas J-L (2016). Chem Mater.

[25] Zang L, Che Y, Moore J S (2008). Acc Chem Res.

[26] Neel A J, Hilton M J, Sigman M S, Toste F D (2017). Nature.

[27] Jurečka P, Šponer J, Černý J, Hobza P (2006). Phys Chem Chem Phys.

[28] Lebègue S, Harl J, Gould T, Ángyán J G, Kresse G, Dobson J F (2010). Phys Rev Lett.

[29] Gould T, Gray E, Dobson J F (2009). Phys Rev B.

[30] Gould T, Simpkins K, Dobson J F (2008). Phys Rev B.

[31] Dobson J F, Gould T, Vignale G (2014). Phys Rev X.

[32] Misquitta A J, Spencer J, Stone A J, Alavi A (2010). Phys Rev B.

[33] Dobson J F (2014). Int J Quantum Chem.

[34] DiStasio R A, von Lilienfeld O A, Tkatchenko A (2012). Proc Natl Acad Sci U S A.

[35] Ambrosetti A, Ferri N, DiStasio R A, Tkatchenko A (2016). Science.

[36] Gobre V V, Tkatchenko A (2013). Nat Commun.

[37] Kim K, Jordan K D (1994). J Phys Chem.

[38] Johnson E R (2011). J Chem Phys.

[39] Maurer R J, Ruiz V G, Tkatchenko A (2015). J Chem Phys.

[40] Christian M S, Otero-de-la-Roza A, Johnson E R (2016). J Chem Theory Comput.

[41] Otero-de-la-Roza A, Johnson E R (2013). J Chem Phys.

[42] Otero-de-la-Roza A, DiLabio G A, Johnson E R (2016). J Chem Theory Comput.

[43] Huang Y, Beran G J O (2015). J Chem Phys.

[44] Řezáč J, Huang Y, Hobza P, Beran G J O (2015). J Chem Theory Comput.

[45] Perdew J P, Burke K, Ernzerhof M (1996). Phys Rev Lett.

[46] Wu Q, Yang W (2002). J Chem Phys.

[47] Wu X, Vargas M C, Nayak S, Lotrich V, Scoles G (2001). J Chem Phys.

[48] Grimme S (2006). J Comput Chem.

[49] Grimme S, Antony J, Ehrlich S, Krieg H (2010). J Chem Phys.

[50] Elstner M, Hobza P, Frauenheim T, Suhai S, Kaxiras E (2001). J Chem Phys.

[51] Becke A D, Johnson E R (2007). J Chem Phys.

[52] Johnson E R (2017). Non-Covalent Interactions in Quantum Chemistry and Physics.

[53] Tkatchenko A, Scheffler M (2009). Phys Rev Lett.

[54] Tkatchenko A, DiStasio R A, Car R, Scheffler M (2012). Phys Rev Lett.

[55] Ambrosetti A, Reilly A M, DiStasio R A, Tkatchenko A (2014). J Chem Phys.

[56] Gould T, Bučko T (2016). J Chem Theory Comput.

[57] Gould T, Lebègue S, Ángyán J G, Bučko T (2016). J Chem Theory Comput.

[58] Dobson J F, Dinte B P, Wang J, Gould T (2000). Aust J Phys.

[59] Dion M, Rydberg H, Schröder E, Langreth D C, Lundqvist B I (2004). Phys Rev Lett.

[60] Vydrov O A, Van Voorhis T (2011). J Chem Phys.

[61] Lee K, Murray É D, Kong L, Lundqvist B I, Langreth D C (2010). Phys Rev B.

[62] Klimeš J, Bowler D R, Michaelides A (2009). J Phys: Condens Matter.

[63] Peng H, Yang Z-H, Perdew J P, Sun J (2016). Phys Rev X.

[64] Zhao Y, Truhlar D G (2008). Theor Chem Acc.

[65] Sun J, Ruzsinszky A, Perdew J P (2015). Phys Rev Lett.

[66] Pernal K, Podeszwa R, Patkowski K, Szalewicz K (2009). Phys Rev Lett.

[67] Hermann J, Tkatchenko A (2018). J Chem Theory Comput.

[68] Kresse G, Furthmüller J (1996). Phys Rev B.

[69] Kresse G, Joubert D (1999). Phys Rev B.

[70] Blochl P E (1994). Phys Rev B.

[71] Bučko T, Lebègue S, Gould T, Ángyán J G (2016). J Phys: Condens Matter.

[72] Klimeš J, Bowler D R, Michaelides A (2011). Phys Rev B.

[73] Román-Pérez G, Soler J M (2009). Phys Rev Lett.

[74] Parrish R M, Burns L A, Smith D G A, Simmonett A C, DePrince A E, Hohenstein E G, Bozkaya U, Sokolov A Y, Di Remigio R, Richard R M (2017). J Chem Theory Comput.

[75] Leconte N, Jung J, Lebègue S, Gould T (2017). Phys Rev B.

[76] Johnson E R, Becke A D, Sherrill C D, DiLabio G A (2009). J Chem Phys.

